# Rare Case of Both Left Atrial and Ventricular Compression by Dissecting Aortic Aneurysm

**DOI:** 10.4274/balkanmedj.2018.0567

**Published:** 2018-09-21

**Authors:** Onur Argan, Dilek Ural, Serdar Bozyel

**Affiliations:** 1 Clinic of Cardiology, Kocaeli State Hospital, Kocaeli, Turkey; 2 Department of Cardiology, Kocaeli University School of Medicine, Kocaeli, Turkey; 3 Clinic of Cardiology, Kocaeli University of Health Sciences, Derince Training Research Hospital, Kocaeli, Turkey

A 75-year-old patient with a history of arterial hypertension was admitted to the emergency service in cardiac arrest. His medical history revealed a thoracic endovascular aortic repair operation that was performed about 2 years ago. According to the information received from relatives, he developed sudden cardiac arrest following chest pain and shortness of breath. The patient’s blood pressure was 70/50 mmHg and the heart rate was 125 pulses/min. ST depression in the D1-AVL-V5-V6 and left ventricular hypertrophy were detected in the electrocardiogram. Parasternal long axis and four-chamber images in the echocardiography showed aortic dissection and compression of the left atrium and the left ventricle caused due to an aneurysm when the patient was evaluated for the first time (Figure 1a). When we repeated the echocardiography before the exitus, the compression of the heart chambers progressed (Figure 1b). Thoracic computed tomography images supported the findings of echocardiography. Compression of the left atrium and ventricle due to 10×11.5 cm descending aorta, including the graft material and the thrombosed pseudolumen, was observed in the computed tomography images (Figure 2a, 2b). The patient with the deep hypotension died shortly after the admission. Written informed consent was obtained from the patient's parents.

Large dissecting aortic aneurysm compressing the heart chambers sufficient to cause hemodynamic deterioration is a rare condition. Studies have reported about the compression related to the right atrium (1), the left atrium (2), and the right ventricle (3) caused by the aortic aneurysm. To our knowledge, this is the first case of both left atrial and left ventricular compression caused due to the dissecting aortic aneurysm. In a patient with aortic dissection, ischemia, aortic valve insufficiency, and tamponade may cause hypotension. The echocardiographic findings of the present case revealed an additional mechanism that may lead to hemodynamic instability. The descending aorta is very near to the left atrium. Compression of the left atrium and ventricle due to the enlarged aorta results in decreased cardiac output and hypotension. The pleural effusion observed in this case suggests the presence of congestive heart failure, which is caused by an increase in the pulmonary capillary wedge pressure due to the compression of the left heart chambers. The thoracic endovascular aortic repair procedure has been used in the treatment of aortic diseases. Although thoracic endovascular aortic repair is clearly less invasive compared with open surgical treatment, it is not free of complications. A mortality rate of 12.9% with endoleak rates of 5.2% and a stent collapse rate of 2.5% have been reported for thoracic endovascular aortic repair (4). These complications can potentially result in severe morbidity and mortality; therefore, early diagnosis and treatment are critical. Computed tomography scan and transthoracic and transesophageal echocardiography are the most common and beneficial diagnostic methods for the aortic diseases. Computed tomography is the most commonly used imaging modality in aortic diseases because of its widespread availability, rapidity, and high sensitivity and specificity. The sensitivity and specificity for diagnosing arch disease are 93% and 98%, respectively, with an overall accuracy rate of 96%. Transthoracic echocardiography is based on determining intimal flaps. The sensitivity and specificity of transthoracic echocardiography have been reported to be 77%-80% and 93%-96%, respectively, in the evaluation of the ascending aorta; however, transthoracic echocardiography is useful in determining a distal dissection of the thoracic aorta in only 70% of patients. Transthoracic echocardiography is limited in patients with obesity, pulmonary emphysema, chest wall disorders, and mechanical ventilation. These limitations have been solved by transesophageal echocardiography. The sensitivity of transesophageal echocardiography reaches 99%, with a specificity of 89%. When mediastinal structures have been compressed by an aortic aneurysm, surgical treatment is mostly recommended (5). Because basically, the exclusion of the aneurysm sac solves the compression findings. The present case reveals an additional mechanism, the “mass affect” that may lead to hemodynamic instability. Due to the huge mass affect, acceleration of surgical treatment can prevent the cardiogenic shock.

## Figures and Tables

**Figure 1:**
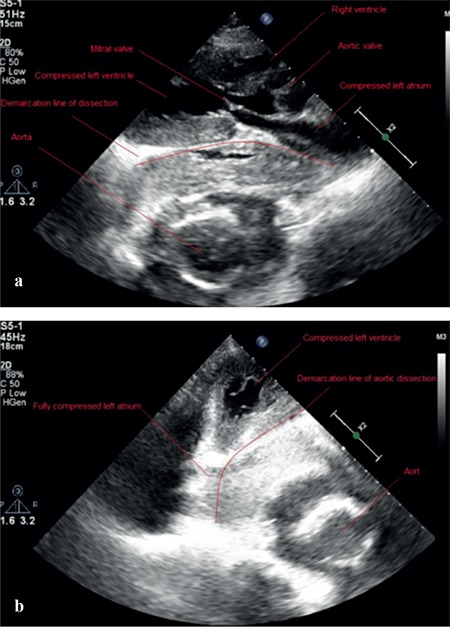
a-b. Long axis view of the transthoracic echocardiographic image showing the dissecting aortic aneurysm causing the compression of the left heart chambers when the patient was evaluated for the first time (a), Four-chamber view of the transthoracic echocardiographic image showing the dissecting aortic aneurysm causing the compression of the left heart chambers before exitus (b).

**Figure 2:**
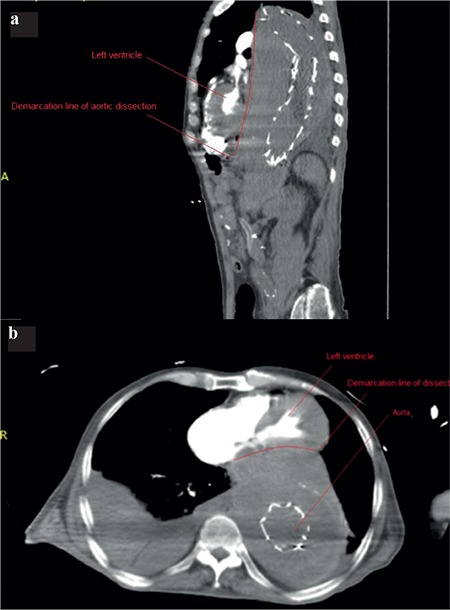
a-b. Sagittal computed tomography image showing the dissecting aortic aneurysm compressing the left heart chambers when the patient was admitted to the hospital (a), Transvers computed tomography image at the level of the ventricle showing the dissecting aortic aneurysm compressing the left heart chambers when the patient was admitted to the hospital (b).
